# Supplementary material for: HIVprotI: an integrated web based platform for prediction and design of HIV proteins inhibitors
Source: J Cheminform. 2018 Mar 9;10:12. doi: 10.1186/s13321-018-0266-y (PMC5845081; doi:10.1186/s13321-018-0266-y)
Supplement: Supplementary file 2 — Additional file 2. Source code of HIVProtI web server. [file 13321_2018_266_MOESM2_ESM.zip › HIVprotI_Source-code/hivprotI/weka-3-6-6/doc/weka/core/logging/OutputLogger.OutputPrintStream.html]

OutputLogger.OutputPrintStream


---


|  |  |  |
| --- | --- | --- |
| **Overview** \| **Package** \| **Class** \| **Tree** \| **Deprecated** \| **Index** \| **Help** \| **Weka's home** | |  |
| **PREV CLASS**   NEXT CLASS | **FRAMES**    **NO FRAMES**     **All Classes** |  |
| SUMMARY: NESTED \| FIELD \| CONSTR \| METHOD | DETAIL: FIELD \| CONSTR \| METHOD |  |


---


## weka.core.logging Class OutputLogger.OutputPrintStream

```
java.lang.Object
  java.io.OutputStream
      java.io.FilterOutputStream
          java.io.PrintStream
              weka.core.logging.OutputLogger.OutputPrintStream
```

**All Implemented Interfaces:**: java.io.Closeable, java.io.Flushable, java.lang.Appendable

**Enclosing class:**: OutputLogger

---

``` public static class OutputLogger.OutputPrintStream extends java.io.PrintStream ```

A print stream class to capture all data from stdout and stderr.

**Version:**
:   $Revision: 4716 $

**Author:**
:   fracpete (fracpete at waikato dot ac dot nz)

---

**Constructor Summary**

`OutputLogger.OutputPrintStream(OutputLogger owner,
java.io.PrintStream stream)`
  
          Default constructor.


| **Method Summary** | |
| --- | --- |
| `void` | `flush()`             ignored. |
| `void` | `print(boolean x)`             prints the given boolean to the streams. |
| `void` | `print(int x)`             prints the given int to the streams. |
| `void` | `print(java.lang.Object x)`             prints the given object to the streams. |
| `void` | `print(java.lang.String x)`             prints the given string to the streams. |
| `void` | `println()`             prints a new line to the streams. |
| `void` | `println(boolean x)`             prints the given boolean to the streams. |
| `void` | `println(int x)`             prints the given int to the streams. |
| `void` | `println(java.lang.Object x)`             prints the given object to the streams (for Throwables we print the stack trace). |
| `void` | `println(java.lang.String x)`             prints the given string to the streams. |

**Methods inherited from class java.io.PrintStream**

`append, append, append, checkError, close, format, format, print, print, print, print, print, printf, printf, println, println, println, println, println, write, write`

**Methods inherited from class java.io.FilterOutputStream**

`write`

**Methods inherited from class java.lang.Object**

`equals, getClass, hashCode, notify, notifyAll, toString, wait, wait, wait`

**Constructor Detail**

### OutputLogger.OutputPrintStream

```
public OutputLogger.OutputPrintStream(OutputLogger owner,
                                      java.io.PrintStream stream)
                               throws java.lang.Exception
```

:   Default constructor.

    **Parameters:**: `owner` - the owning logger: `stream` - the stream **Throws:**: `java.lang.Exception` - if something goes wrong


**Method Detail**

### flush

```
public void flush()
```

:   ignored.

    :   **Specified by:**: `flush` in interface `java.io.Flushable` **Overrides:**: `flush` in class `java.io.PrintStream`

---


### print

```
public void print(int x)
```

:   prints the given int to the streams.

    :   **Overrides:**: `print` in class `java.io.PrintStream`
    :   **Parameters:**: `x` - the object to print

---


### print

```
public void print(boolean x)
```

:   prints the given boolean to the streams.

    :   **Overrides:**: `print` in class `java.io.PrintStream`
    :   **Parameters:**: `x` - the object to print

---


### print

```
public void print(java.lang.String x)
```

:   prints the given string to the streams.

    :   **Overrides:**: `print` in class `java.io.PrintStream`
    :   **Parameters:**: `x` - the object to print

---


### print

```
public void print(java.lang.Object x)
```

:   prints the given object to the streams.

    :   **Overrides:**: `print` in class `java.io.PrintStream`
    :   **Parameters:**: `x` - the object to print

---


### println

```
public void println()
```

:   prints a new line to the streams.

    :   **Overrides:**: `println` in class `java.io.PrintStream`

---


### println

```
public void println(int x)
```

:   prints the given int to the streams.

    :   **Overrides:**: `println` in class `java.io.PrintStream`
    :   **Parameters:**: `x` - the object to print

---


### println

```
public void println(boolean x)
```

:   prints the given boolean to the streams.

    :   **Overrides:**: `println` in class `java.io.PrintStream`
    :   **Parameters:**: `x` - the object to print

---


### println

```
public void println(java.lang.String x)
```

:   prints the given string to the streams.

    :   **Overrides:**: `println` in class `java.io.PrintStream`
    :   **Parameters:**: `x` - the object to print

---


### println

```
public void println(java.lang.Object x)
```

:   prints the given object to the streams (for Throwables we print the stack
    trace).

    :   **Overrides:**: `println` in class `java.io.PrintStream`
    :   **Parameters:**: `x` - the object to print


---


|  |  |  |
| --- | --- | --- |
| **Overview** \| **Package** \| **Class** \| **Tree** \| **Deprecated** \| **Index** \| **Help** \| **Weka's home** | |  |
| **PREV CLASS**   NEXT CLASS | **FRAMES**    **NO FRAMES**     **All Classes** |  |
| SUMMARY: NESTED \| FIELD \| CONSTR \| METHOD | DETAIL: FIELD \| CONSTR \| METHOD |  |


---
